# Cytolethal Distending Toxin Subunit B: A Review of Structure–Function Relationship

**DOI:** 10.3390/toxins11100595

**Published:** 2019-10-12

**Authors:** Benoît J. Pons, Julien Vignard, Gladys Mirey

**Affiliations:** 1 Toxalim (Research Centre in Food Toxicology), Université de Toulouse, INRA, ENVT, INP-Purpan, UPS, 31300 Toulouse, France; benoit.pons@inra.fr (B.J.P.); julien.vignard@inra.fr (J.V.); 2 Research Centre in Food Toxicology, Université Toulouse III–Paul Sabatier (UPS), 31400 Toulouse, France

**Keywords:** cytolethal distending toxin, CdtB subunit, structure-function relationship, key residues

## Abstract

The Cytolethal Distending Toxin (CDT) is a bacterial virulence factor produced by several Gram-negative pathogenic bacteria. These bacteria, found in distinct niches, cause diverse infectious diseases and produce CDTs differing in sequence and structure. CDTs have been involved in the pathogenicity of the associated bacteria by promoting persistent infection. At the host-cell level, CDTs cause cell distension, cell cycle block and DNA damage, eventually leading to cell death. All these effects are attributable to the catalytic CdtB subunit, but its exact mode of action is only beginning to be unraveled. Sequence and 3D structure analyses revealed similarities with better characterized proteins, such as nucleases or phosphatases, and it has been hypothesized that CdtB exerts a biochemical activity close to those enzymes. Here, we review the relationships that have been established between CdtB structure and function, particularly by mutation experiments on predicted key residues in different experimental systems. We discuss the relevance of these approaches and underline the importance of further study in the molecular mechanisms of CDT toxicity, particularly in the context of different pathological conditions.

## 1. Introduction

The Cytolethal Distending Toxin (CDT) is a bacterial toxin first identified in the late 1980s by Johnson and Lior in some *Escherichia coli* strains, *Shigella* and *Campylobacter* spp [1,2,3,4]. One decade later, several other Gram-negative pathogenic bacteria were found to produce or carry the CDT genes. These include *Haemophilius ducreyi*, a pathogen causing human sexually transmitted disease [5], *Aggregatibacter actinomycetemcomitans,* involved in the pathogenesis of periodontis [6,7], and *Helicobacter* spp, implicated in several chronic infections [8]. Today, more than 30 Proteobacteria have been found to harbor the CDT genes (reviewed in [9,10]). CDT is a tripartite toxin composed of three subunits, CdtA, CdtB and CdtC, encoded by the eponym genes [11]. CDT functions as an AB_2_ heterotrimeric toxin with two regulatory subunits (CdtA and CdtC) responsible for the transport of the active subunit (CdtB), which bears the catalytic activity [12,13]. The typhoid toxin produced by *Salmonella enterica serovar Typhi* also contains a CdtB subunit but has a different A_2_B_5_ structure—a pentameric disc of five PltB regulatory subunits and two active subunits, CdtB and PltA, with CdtB sustaining the pathogenicity of the toxin [14].

The nomenclature used here follows the recommendations of Jinadasa and collaborators: Aact refers to *A. actinomycetemcomitans*, Cjej to *Campylobacter jejuni* CDT, Hduc to *H. ducreyi*, Hpar to *Haemophilius parasuis*, Hhep to *Helicobacter hepaticus* and Styp to *S. Typhi* [9]. EcolCDT-I and EcolCdtB-I refer, respectively, to *E. coli* CDT-I and the associated CdtB. It should be noted that *cdt-II* gene-positive *E. coli* were recently found to be *Escherichia albertii* [15]. For clarity, however, we chose to use the previous label: EcolCDT-II and EcolCdtB-II.

### 1.1. Cellular Effects of CDT

CDT was named for its ability to induce cell distension and death [1]. CDT-dependent cell death results from an apoptotic response, either in fibroblast, epithelial or lymphoblastoid mammalian cells [16,17]. Cell distension can be indicative of premature cellular senescence [18]. Furthermore, CDT intoxication leads to cell cycle arrest in G2/M [19,20] and in G1/S in certain cell lines, probably depending on their p53 status [21,22]. Such cellular outcomes are hallmarks of DNA Damage Response (DDR) activation. Noticeably, CDT can induce DNA lesions in intoxicated cells [23], first characterized as double-strand breaks (DSB) [24]. Further investigations demonstrated that direct DSB were mainly observed after exposure to high concentrations of CDT, whereas more moderate doses primarily induce single-strand breaks (SSB), which subsequently degenerate into DSB upon replication [25]. CDT activates the replicative stress or DSB-related branches of the DDR [26,27]

In addition, the lymphoblastoid Jurkat cell line exposed to AactCDT presents a reduced level of phosphatidylinositol-3,4,5-trisphosphate (PIP_3_), which is associated with an increase of phosphatidylinositol-3,4-bisphosphate (PI3,4P_2_) and an alteration of the phosphatidylinositol-3-kinase (PI-3K) signaling pathway involved in cell survival and proliferation [28]. These findings have already been extensively reviewed [9,10,29] and will not be detailed in this manuscript.

### 1.2. CDT Acts as A Virulence Factor

Several studies have shown that CDT is important for bacterial pathogenicity and inflammation in mice or rat models [30,31,32]. Although these studies showed no obvious effect of the CDT toxin at early infection points, HhepCDT was shown to promote persistent colonization in mice gut [33,34]. CDT-induced inflammation can also occur in vitro, as assessed by the production of proinflammatory cytokines in many cell lineages [18,35,36].

Two factors might explain the pathogenicity of CDT. First of all, CDT appears to mainly target the immune system. Lymphocytes are extremely sensitive to CDT-mediated cell cycle arrest and death [21,37,38]. Macrophages are less sensitive than lymphocytes to CDT treatment [39,40]. However, macrophages produce inflammatory cytokines, show decreased phagocytosis capacity, and are more prone to autophagy after CDT treatment [41,42,43]. Secondly, the capacity of CDT to induce cell death leads to epithelial barrier permeabilisation. AactCDT application on ex vivo gingival tissue induces cell junction dissolution, detachment of the keratinized outer layer [44], and adherence junction remodeling [45]. Taken together, epithelial barrier disruption and immune system sensitivity may help CDT-producing bacteria to better colonize host-tissues while protecting themselves from the immune system.

In addition to its contribution to chronic infection and inflammation, CDT involvement in carcinogenesis has been shown on several levels. In human colorectal cancer (CRC) patients, CDT-producing bacteria can be found around tumors but not in normal parts of the colon [46]. Only bacteria bearing the whole CDT operon are able to induce dysplasia and carcinogenesis in murine models [47,48,49]. Finally, chronic infections of rat embryonic fibroblasts or normal human colon epithelial cells with CDT promote the acquisition of malignant transformation hallmarks [50,51], which are exacerbated in isogenic cell lines mimicking the mutations of genes found in CRC models.

### 1.3. CDT Journey: From the Bacteria to Host-Cell Nucleus

In order to be excreted by the bacteria, each of the three CDT subunits possesses an N-terminal amino acid secretion signal [52]. The subunits are then addressed to periplasm, where the holotoxin is reconstituted [53] before excretion in the culture supernatant [54] or through outer membrane vesicles (OMV) [55]. Despite its different subunit organization in holotoxin, StypCdtB also includes a secretion signal required for toxin excretion [56] and bacterial toxicity [57].

After CDT secretion, CdtA and CdtC are mainly responsible for host-cell recognition, by binding to the plasma membrane [58,59]. This binding involves cholesterol-rich lipid rafts, the sphingomyelin synthase 1 (SGMS1) protein, and glycoproteic or glycolipidic cellular receptors that differ between the CDTs [60,61]. Although membrane binding of CDT is mostly attributed to CdtA and CdtC, one study has hypothesized that AactCdtB may also be able to bind cholesterol [62].

Upon membrane binding, CdtA remains at the plasma membrane, while CdtB and CdtC are internalized [63,64]. CdtB and CdtC are endocytosed [65,66], and only CdtB reaches the nucleus. Of note, three putative nuclear localization signals (NLS) involved in CdtB transfer to the nucleus have been identified [67,68]. CdtB nuclear localization involves a retrograde transport through the endoplasmic reticulum and the trans-Golgi network [69,70]. This was reviewed by Frisan [71], but the exact intracellular pathway might differ for toxins of different bacterial strains [70].

## 2. Sequence and 3D Structure Similarities

**Figure 1:**
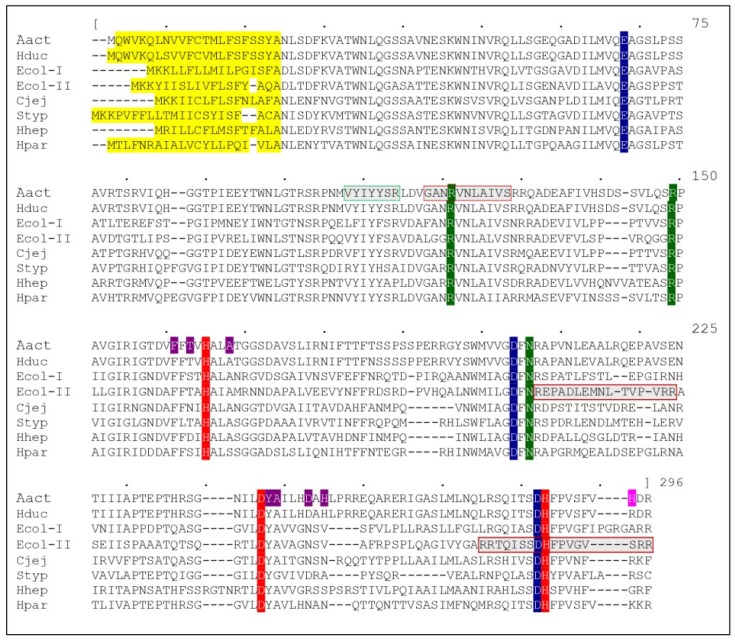
Sequence alignment between CdtBs from different organisms: AactCdtB, HducCdtB, EcolCdtB-I, EcolCdtB-II, CjejCdtB, StypCdtB, HhepCdtB and HparCdtB (Genbank accession numbers: BAA33486.1, AAB57726.1, AAD10622.1, AAA18786.1, AAB06708.1, CAD02120.1, AAF19158.1 and ACL31958.1 respectively). Alignment was obtained with MUSCLE software and the upper numbering refers to the consensus sequence (not presented). Catalytic residues are represented in red, metal binding residues in blue and substrate binding residues in green. In AactCdtB, residues potentially involved in phosphatase activity are in purple, while the H281 activating residue is in pink. Several putative regions are represented: secretion signal in yellow, nuclear localization signal in gray with a red square and cholesterol recognition sequence in gray with a green square.

CdtB is the most conserved subunit among the different CDTs, with a percent amino acid identity of at least 48% (Figure 1). On the other hand, CdtA and CdtC are less conserved, with a minimum percent amino acid identity of 26% and 19%, respectively [72]. Interestingly, the HducCDT and AactCDT holotoxins exhibit an extensive similarity with a percent amino acid identity of more than 90% overall, CdtB being the most conserved, with a percent amino acid identity of 97%.

The first analysis of the CdtB sequence revealed no homology to any known proteins [11,52]. In the early 2000s, the emergence of new protein sequence analysis software made it possible to relate CdtB to metalloenzymes with distinct activities. The *psi-blast* software revealed that bovine DNase I sequence was close to EcolCdtB-II [73], and the same homology was observed for CjejCdtB using the *clustalW* software [12]. The matrix scores between EcolCdtB-II and DNase I were very close to those between EcolCdtB-I and EcolCdtB-II or EcolCdtB-III, which supported the closeness with DNase I [73]. The overall percent amino acid identity between HducCdtB and bovine DNase I is 12% [13] and essential residues for DNase I activity [74] are well conserved. Conversely, using *probe* and *psi-blast* software to find analogous proteins to bovine DNase I, HducCdtB was identified as a member of the endonuclease-exonuclease-phosphatase (EEP) family [75], which includes other nucleases such as APEX1 (human Apurinic/Apyrimidinic Endodeoxyribonuclease 1) or ExoIII (Exonuclease III of *E. coli*) but also lipid phosphatases. For example, inositol polyphosphate 5-phosphatase domain (IP5P) of synaptojanin from *Schizosaccharomyces pombe* has been shown to present sequence homology with HhepCdtB [76] and AactCdtB [77].

**Figure 2:**
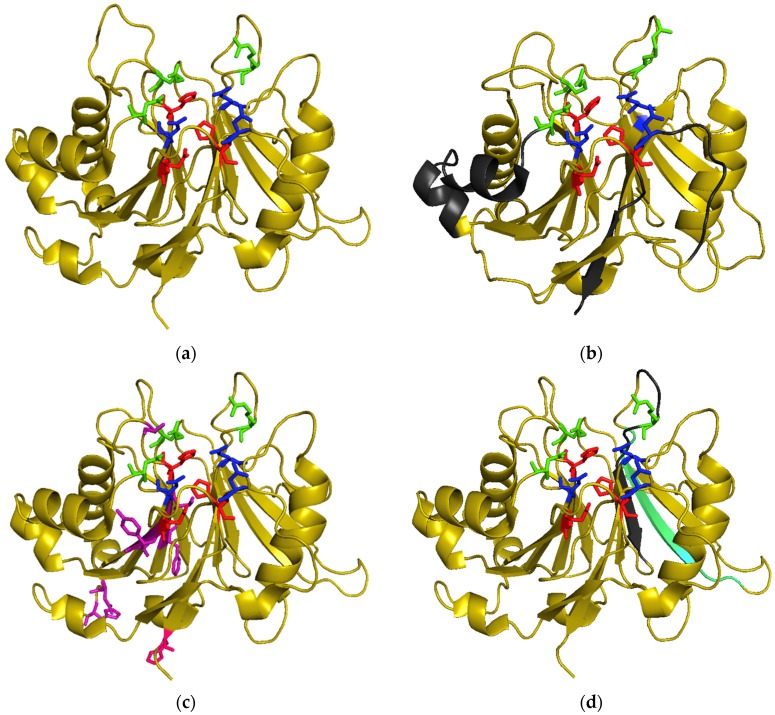
3D structure of CdtBs: catalytic residues are represented in red, metal binding residues in blue and substrate binding residues in green. (**a**) HducCdtB (PDB accession number: 1sr4); (**b**) EcolCdtB-II (PDB accession number: 2f1n) with putative NLS regions in grey; (**c**) AactCdtB (PDB accession number: 2f2f) with residues potentially involved in phosphatase activity in purple and the H281 activating residue in pink; (**d**) AactCdtB (PDB accession number: 2f2f) with putative nuclear localization signal region in grey and cholesterol recognition sequence in cyan.

The first CDT crystallization was performed by Nesic and collaborators on HducCDT, whose subunits were separately purified under denaturing conditions and co-refolded as holotoxin [13]. The 3D-structure revealed that CDT subunits adopt the AB_2_ structure via three interdependent protein-protein interfaces involving globular and non-globular interactions. CdtA and CdtC share structural homologies with lectin-type proteins and form a large aromatic patch and a deep groove at the surface of the holotoxin. This is probably important for host membrane binding. The similarity between CdtB and bovine DNase I was confirmed by structure alignment. Indeed, despite the weak overall sequence similarity between CdtB and DNase I, residues involved in DNase I catalysis are well conserved in CdtB (see later). Moreover, CdtB exhibits the four-layered fold that is characteristic of DNase-like proteins: stranded β-sandwich flanked with α-helix and loops (Figure 2). As previously noted, the differences between CdtB and DNase I lay on the number of strands in the β-sheets: six strands twice for DNase I, six strands on one side and eight on the other side for CdtB [78].

The 3D structure permits the identification of several regions of interaction between CdtB and CdtA or CdtC [72,79]. The N-terminal domain of CdtC covers the CdtB active site with several strong interactions. CdtC D22 residue forms a salt bridge with CdtB R139, while CdtC L23 has van der Waals interactions with both E60 and H155 from CdtB [72]. Interestingly, the CdtC N-terminal tail binds several residues directly involved in CdtB catalytic activity: CdtC D31 interacts with CdtB R177 and R144, while CdtC V32 is in contact with CdtB, E66 and H160. These interactions have been shown to inhibit CdtB in the holotoxin context, potentially limiting its catalytic activity to host cell-internalized CdtB [13]. Furthermore, the HducCdtB 261-272 (especially R269 making hydrogen bonds with CdtA S166 and CdtC R39) and HducCdtB 41-54 (particularly N46 and R50, in hydrophobic interaction with CdtC P119) domains are also involved in the regulatory subunits interactions [79].

The AactCDT structure was determined from a recombinant holotoxin directly purified under native conditions [80]. As expected on the basis of sequence similarities, the 3D structure of AactCDT is quite similar to HducCDT, particularly for CdtB subunits. The ternary and quaternary structures are almost identical (Figure 2), with a root-mean-square-deviation (RMSD) of 0.71 Å between these CDT holotoxins and 0.30 Å between CdtBs. Most of the residues showing differences between AactCDT and HducCDT are located on the protein surface. There are, however, differences in toxin aggregation, as AactCDT holotoxin is found in a dimeric form within the crystal. Finally, structure alignments further support the similarity between AactCdtB and IP5P active sites [10].

The structure of the isolated CdtB from EcolCDT-II was also resolved [81] and showed a good conservation of active site residues and overall structure with HducCdtB and AactCdtB [78]. Indeed, RMSD between HducCdtB and EcolCdtB-II structures is 1.02 Å, with a percent amino acid identity of 48%. However, some discrepancies exist between EcolCdtB-II and the two other toxins structures, particularly for backbone regions involved in CdtA and CdtC interaction. It is not clear if those variations are due to intrinsic differences between these toxins or due to the absence of CdtA and CdtC subunits in the EcolCdtB-II crystal.

The crystal structure of the whole typhoid toxin from S. Typhi was solved, indicating that StypCdtB structure aligned very well to HducCdtB, especially for active site residues: RMSD was 0.947 Å, with a percent amino acid identity of 52% [14]. In contrast with hydrogen and van der Waals interactions found between the different CDT subunits, StypCdtB is fused to the PltA subunit by a disulfide bond between CdtB C269 and PltA C214.

To conclude, the CdtB sequence and structure appear to be well conserved between the different producing bacteria. They also exhibit active site similarities with EEP enzymes involved in DNA or phospholipid catabolism.

## 3. CdtB: One Active Site, Two Enzymatic Activities

Because of its sequence and structure similarities with DNase I, it has been proposed that CdtB possesses nuclease activity. DNase I is a divalent metal ion-dependent phosphodiesterase that catalyzes the degradation of double-stranded DNA through the formation of multiple SSBs [82]. Such activity is easily measurable by a classic plasmid digestion assay [83]. Therefore, this test has been adapted to study CdtB nuclease activity [73] and has been widely used. However, several reports emphasized the extremely weak plasmid degradation activity of CdtB compared to DNase I [84,85,86,87]. Huge CdtB concentrations and long incubation times were required to assess its nuclease activity. Under these experimental conditions, HducCdtB with deleted catalytic region, or even non-nucleases HducCdtA and HducCdtC, have been shown to induce plasmid degradation [88,89]. We previously reported that recombinant Ecol-CdtB and HducCdtB mutated in putative catalytic residues were not affected in their capacity to degrade plasmid DNA, while they did not induce any DNA damage in exposed cells compared to their wild type counterparts [89]. This suggests that the classic in vitro digestion assay may not be suited to study CdtB nuclease activity. The lack of observable activity has not yet been explained—it could be caused by intrinsic differences between plasmid DNA and chromatin, which is the usual CdtB substrate, or by the need of a host-cell partner such as a protein. Therefore, until a more suitable biochemical assay is developed, the CdtB nuclease activity could instead be estimated in cellular systems, either by direct assessment of DNA breaks with pulse-field gel electrophoresis [24], comet assay [25,26], or by monitoring the DDR activation [18,23,25,26,27,89,90]. CdtB certainly exhibits a DNA damaging ability but its exact biochemical activity differs from DNase I and has yet to be fully characterized.

On the basis of structural and sequences similarities with various lipid-phosphatases such as synaptojanin, AactCdtB has also been described to dephosphorylate PIP_3_ [77]. In vitro phosphate release was specifically observed when incubating AactCdtB with PIP_3,_ but no other tested phospholipid substrate. This activity was sensitive to the presence of phosphatase inhibitors. CdtB exhibits a similar yet reduced PIP_3_ degradation compared to Phosphatase and TENsin homolog (PTEN), a well characterized human PIP_3_ phosphatase. However, in contrast to PTEN, which dephosphorylates PIP_3_ in position 3 to form PI4,5P_2_, CdtB specifically removes the phosphate in 5-position and produces PI3,4P_2_. The PIP_3_ level decreases in Jurkat T cells treated with AactCDT, in a dose and time dependent manner. This is accompanied by an increase in the PI3,4P_2_ level, but not the PIP4,5P_2_ level, which confirms this difference between CdtB and PTEN [28]. Therefore, CdtB seems to function as a phosphatidylinositol 5-phosphatase, comparably to human SHIP1 and SHIP2 proteins [91]. The cellular PIP_3_ depletion could have consequences on downstream targets of PIP_3_, notably in the PI-3K pathway, as Jurkat cells exhibit reduced phosphorylated AKT and GSK3β levels upon AactCDT treatment [28].

In order to decipher the importance of each activity in the CDT-mediated cellular or pathologic outcomes, considering separately the biological significance of the nuclease and phosphatase activities is of great relevance. As mentioned above, nucleases and phosphatases from the EEP family display sequence similarities with conserved catalytic residues [75]. Therefore, the putative nuclease and phosphatase activities of CdtB may share the same active site. Moreover, predictive models of CdtB interaction with DNA or phospholipid indicate that the same amino acids are involved in binding both substrates [13,28]. Consequently, attempts to uncouple DNase and phosphatase activities of CdtB by functional analyses of mutants have been difficult and somewhat inconclusive to date.

## 4. Structure–Function Relationship

Based on the sequence and structure comparisons with DNase I and phosphatases, several studies reported the characterization of many CdtB mutants, either biochemically or in living cells. Here, we review the structure–function relationships found for different CdtB mutants, including discrepancies between in vitro tests from different publications or between in vitro and cellular effects. The mutations and residues are classified by their reported or hypothesized roles, most of which are based on comparison with other proteins. All mutants and reported phenotypes are listed in Table 1.

### 4.1. Catalytic Residues

The functions of several CdtB key residues were originally derived from sequence comparison of EcolCdtB-II with bovine DNase I. Two DNase-I histidines (H134 and H252) involved in phosphodiester bond hydrolysis are conserved in EcolCdtB-II (H154 and H261) alongside their hydrogen bond pairs: E78 and D212 in DNase I interact with H134 and H252 respectively, and are aligned to E86 and D229 in EcolCdtB-II. Substitution of each of these residues with alanine resulted in a loss or reduction of nuclease activity in the plasmid digestion assay, as well as cell distension and cell cycle arrest, thus confirming their importance in CdtB activity [73]. Yet, only three of these residues were subsequently confirmed to be relevant for catalytic activity, according to structural comparison between crystalized bovine DNase I and HducCdtB [13,72]. Indeed, the 3D structure showed that DNase I E78 aligns with HducCdtB V118 residue, rather than E86, as previously anticipated by sequence analysis. This is merely a position alignment, since V118 does not seem to interact with the catalytic H160 of HducCdtB in the 3D structure. Interestingly, if V118 is replaced by a glutamate as in DNase I, HducCdtB completely loses biochemical and cellular activities. Therefore, V118 was not considered as a catalytic residue and has not been further studied. This also indicates structural and functional divergences between DNase I and CdtB, which might be correlated with differences observed in plasmid digestion assay.

Mutations of the three catalytic residues mentioned above were widely used in the literature to abolish the catalytic activity of CdtB. First of all, proliferating cells exposed to a toxin bearing a CdtB mutated in the corresponding DNase I H134 catalytic residue do not suffer DNA damage, cell cycle block or apoptosis, as shown with EcolCDT-I and-II [25,27,51,73,88,89], CjejCDT [12,93], AactCDT [40,64,77] and HparCDT [94]. However, this AactCDT mutant still induces apoptosis in non-proliferating blood cells [40], suggesting that CdtB catalytic activity may not be involved in the observed toxicity. Several authors also reported a suppression of DNA damage induction for this mutation upon direct CdtB expression or delivery in mammalian cells [12,57,89] or yeasts [92]. A loss of function was also observed for H160Q StypCdtB compared to the WT, both in cellular tests with a chimeric toxin CjejCdtA-StypCdtB-CjejCdtC [57], and in the context of the typhoid toxin in a murine infection model [14]. Nevertheless, discrepancies have been reported in biochemical studies. The plasmid digestion ability was reported to be abolished for EcolCdtB-II H154A [73,84] and HparCdtB H161Q [94] but unchanged for EcolCdtB-I H153A [89]. Concerning AactCdtB, the H160Q mutation diminished both plasmid and PIP_3_ in vitro degradation [77], while H160G greatly increased the phosphatase activity without altering the nuclease activity [40].

In the same way, mutation on the second catalytic histidine (H252 in DNase I) abolishes most of the activity. H261A mutation in EcolCdtB-II [73] and similar substitutions in AactCdtB [77,94,95,96] or HhepCdtB [36,76,99] greatly impaired cellular effects in yeast, mammalian cells, ex vivo models and xenograft mice. In vitro plasmid digestion was completely suppressed for EcolCdtB-II H261A [73] and HhepCdtB H265L [99] but remained unchanged for AactCdtB H274Q [77]. This same mutant was also reported to exhibit a reduced PIP_3_ dephosphorylation activity. Lastly, the aspartate homologous to DNase I D212 is also crucial for the toxin activity. The D229A mutation in EcolCdtB-II inhibits in vitro plasmid degradation, cell distension and cell cycle block [73]. An analog mutation in CjejCdtB prevents cell cycle block and DNA damage induction in yeast [95] and in primary human fibroblasts [96].

### 4.2. Metal Binding Residues

Due to sequence similarity with Mg^2+^-dependent enzymes [75], it has also been proposed that CdtB activity depends on divalent cation. Similarly to the catalytic amino acids, three metal binding residues were first hypothesized based on sequence alignment with DNase I [73], which was further supported by structural comparison [13]. For CjejCDT [12], StypCDT [57] and AactCDT [77], the homolog of the DNase I metal binding D168 has been shown to be involved in chromatin condensation upon plasmid transfection and cell cycle block by holotoxin or chimeric toxin. In HducCDT, coupling this mutation to the mutation of a catalytic histidine also suppressed CDT-induced cell cycle block and in vitro plasmid degradation [13]. In a similar way to catalytic histidine mutation, AactCDT D199G [40] lost its ability to induce apoptosis in proliferating blood cells but not in non-proliferating ones. Finally, PIP_3_ dephosphorylation and plasmid degradation ability were impaired in both D199S [77] and D199G [40] mutants of AactCdtB. A second metal binding residue, aligned with D251 of DNase I, is essential for EcolCDT-II [73] and HducCDT [69,89,100,101] to induce cell cycle block and DNA damage. However, while substituting this residue with arginine has been shown to impede plasmid degradation for EcolCdtB-II [84], the corresponding mutant in HducCdtB did not seem affected in a similar assay [89]. As expected, the D199A-D273A double substitution of these metal binding residues also inhibited cell cycle arrest and cell death induction by AactCDT [64]. On the other hand, the last conserved metal binding residue, homologous to E39 in DNase I, is not essential for CdtB activity as the HhepCdtB E60V mutation does not alter plasmid degradation or holotoxin-induced cellular death [76].

Finally, the N27 residue of HhepCdtB was described as a potential metal binding residue on the basis of a sequence alignment with the IP5P domain of synaptojanin from *S. pombe* but does not seem to be involved in CdtB-induced plasmid digestion or apoptosis [76].

### 4.3. Substrate Binding Residues

Along with catalytic and metal binding residues, sequence comparison revealed that EcolCdtB-II R123 and N194 are homologous to R111 and N170 DNA binding residues of DNase I [73]. Structural alignment corroborated this prediction for the EcolCdtB-II N194, but not for R123. However, two other arginines were speculated to bind DNA: R144 and R117 in HducCdtB, corresponding to R41 and R111 in DNase I [13,72]. Sequence alignment showed that the binding triad of DNase I and AactCdtB is found in the IP5P domain of *S. pombe* synaptojanin and authors suggested that these amino acids might also be involved in lipid binding [28].

Substitution to alanine of any or all of these three residues resulted in highly impaired cell cycle arrest and inflammation mediated by HducCDT [13] or AactCDT [28,42,77]. The associated mutations of AactCdtB Q35, N201 and Y239, three residues predicted to be involved in DNA contact, prevented AactCDT-induced cell cycle and proliferation defects without altering the CdtB nuclear localization [64]. As for functional analyses of catalytic and metal binding residues, the measure of nuclease activity through plasmid degradation of CdtB substrate binding mutants generated contradictory results. Some showed decreased, increased or unaltered activity compared to WT [13,28,42,77]. Intriguingly, while the HducCdtB N201A mutant displayed no activity in the plasmid digestion activity, supplementary mutation of the two other substrate binding residues, R117 and R144, restored DNase activity and increased it up to 3-fold higher than the WT CdtB [28]. All tested substrate binding mutants exhibited a reduced phosphatase activity, which appears to show that these amino acids are not only essential for binding to DNA but also for lipid substrates binding [28,77].

### 4.4. Residues Potentially Involved in Phosphatase Activity

Sequence comparison highlighted residues conserved between AactCdtB and IP5P domain of *S. pombe* synaptojanin but not with DNase I, suggesting that this domain might be involved specifically in the putative phosphatase activity [28]. Docking simulations highlighted putative interactions between A163 of AactCdtB and inositol-3,4,5-trisphosphate or inositol-3,4-bisphosphate, corresponding to the sugar part of the CdtB hypothetical substrate and product, respectively. The A163R substitution reduced AactCdtB DNase and phosphatase activities in vitro and impaired CDT-induced cell cycle arrest in Jurkat cells but to a lesser extent compared to substrate binding mutants. Conversely, the cell cycle of HeLa cells treated with this mutant was blocked comparably to WT AactCDT-treated cells, although the absence of DNA damage induction should be noted. This is inconsistent with previous findings demonstrating that CDT-mediated cell cycle arrest depends on ATM, the major kinase governing the cellular response to DSBs [21].

Six other residues were selected because they were close to catalytic H160 or H274 residues and conserved in AactCdtB and IP5P, but not DNase I [28]. Three independent double mutants were generated to match DNase I sequence F156I-T158I, Y239R-A240I and D244G-H246L. The F156I-T158I mutation impaired Jurkat cell cycle arrest and decreased in vitro phosphatase activity but enhanced plasmid digestion. Triple mutant F156I-T158I-A163R presented similar plasmid digestion activity compared to the double mutant F156I-T158I, despite a null DNase activity observed for the A163R mutant. The Y239R-A240I and D244G-H246L double mutants were unable to digest DNA, dephosphorylate PIP_3_ or block Jurkat cell cycle progression. Surprisingly, the quadruple mutant Y239R-A240I-D244G-H246L was defective in phosphatase activity and cell cycle arrest induction but presented an increased in vitro nuclease activity. While Y239 and A240 are close to the catalytic site, according to AactCdtB structure (Figure 2), D244 and H246 seem more distant. As for T158, D244 and H246 are not conserved among the different CdtBs, preventing extrapolation of these results.

### 4.5. Mutations Identified in Patients Isolates

Two natural mutants of AactCdtB were identified in bacterial isolates from patient periodontitis. The *cdtB* gene of an isolate presenting a very high distending capacity carried the H281R substitution [87]. Recombinant AactCdtB H281R exhibited an improved plasmid and chromatin degradation, and the corresponding holotoxin induced more distension and cell cycle arrest in host cells. Remarkably, arginine in position 281 actually represents the natural sequence of HducCdtB, corresponding to one of only nine residues that are not strictly identical in the CdtB sequences of these two bacteria. The function of H281 was further validated with other mutations. While H281K increased cellular defects compared to WT AactCDT, the H281D mutant exhibited reduced distending and blocking capacities that were completely abolished after H281A mutation, although this mutant was poorly affected during in vitro plasmid digestion assays. Taken together, these data show that H281 plays a role in CdtB activity. On the other hand, a naturally truncated variant of AactCdtB has been identified in two patients with chronic periodontitis [88]. This mutant lacks the 116-188 residues, including the catalytic H160. It cannot form a holotoxin with CdtA and CdtC, and is therefore unable to induce DNA damage or cytotoxicity. However, this mutant presented a higher nuclease activity, as assessed by in vitro plasmid digestion.

### 4.6. Cholesterol Binding Domain

A sequence analysis of AactCdtB revealed the presence of a potential cholesterol recognition amino-acid consensus sequence (CRAC site) [62], the VYIYYSR residues in position 104-110. Substitution for a proline of V104, Y105 and Y107 reduced in vitro cholesterol binding but not PIP_3_ dephosphorylation activity. It also suppressed cellular binding, cellular internalization, cell-cycle block and apoptosis. Interestingly, the R110P substitution increased cholesterol-binding capacity without altering phosphatase activity and cellular defects. Thus, the authors proposed that AactCDT association with host-cells is dependent on both CdtB and CdtC.

### 4.7. Nuclear Localization Signals

In AactCdtB, the 48-124 region was identified as NLS by monitoring intracellular localization of several microinjected CdtB deletion mutants and subsequent cell death [67]. Replacement of the 48-124 residues with a classical monopartite SV40 T NLS was able to restore both CdtB function and localization. This putative NLS was further refined to residues 114-124 [67], which are also required to mediate cytotoxicity in yeast [92]. Two arginines directly following this NLS were mutated in AactCDT (R125S-R126T) in order to recapitulate the effect of the AactCdtBΔ114-124 mutant. However, neither CdtB localization nor apoptosis induction were disturbed, although cell cycle defects were reduced compared to WT AactCDT [64].

Two other putative NLS were identified in EcolCdtB-II by sequence analysis: the 195-210 and 253-269 regions [68]. Deletion mutants of one or both of these NLS did not impair in vitro plasmid digestion or host-cell membrane binding, but their respective toxicities toward host-cells were different. While mutants lacking the 195-210 peptide were devoid of any cytotoxic effects when directly delivered in mammalian cells upon electroporation, the EcolCdtB-IIΔ253-269 mutant induced defects similar to WT, although lacking catalytic H260 and metal binding D261 residues. However, reconstituted holotoxins deleted for any of these sequences were unable to induce cell cycle arrest, probably due to impaired nuclear localization: EcolCdtB-IIΔ253-269 showed a diffuse cytoplasmic localization, whereas EcolCdtB-IIΔ195-210 concentrated at the nuclear periphery. These differences suggest that the 195-210 region acts as a typical NLS while the 253-269 residues are involved in cellular trafficking.

The NLS identified in AactCdtB and EcolCdtB-II are not related. As shown in Figure 2, the EcolCdtB-II NLS are located on the exterior of the protein while the AactCdtB NLS is mainly contained in one β-strand in the center of the structure. This divergence is not explained, but could mean a different mode of nuclear transport between CdtBs from various origins and might be due to limited sequence similarities between CdtB subunits (Figure 1). Except for EcolCdtB-II, the AactCdtB putative NLS is mainly conserved but has not been validated in other CDTs. On the other hand, the EcolCdtB-II NLS are located in regions with high diversity. Nonetheless, Damek Paprowa and collaborators mutated arginines R189 and R190 in AacCdtB, supposedly corresponding to the end of the 195-210 EcoCdtB-II NLS [64]. The resulting mutant localization was indeed disturbed and the cellular defects induced by the toxin were diminished at the same time, compared to WT AactCDT. However, it appears that the two arginines mutated in this study do not align with EcolCdtB-II NLS (Figure 1). Therefore, conservation of the EcolCdtB-II NLS in AacCdtB seems unlikely.

## 5. Conclusions

Understanding the role of CDT in the physiopathology of CDT-producing bacteria is of crucial interest and needs deeper investigations. The parallel study of different CDTs allows us to highlight their common characteristics, but also underlines their specific properties. This is particularly relevant to CDT, found in bacteria present in different physiological niches and involved in different pathologies. While the regulatory moiety of CDT should dictate the cell type targeting and thus susceptibility, the CdtB catalytic subunit sustains the toxic activity whose molecular basis still needs to be clarified.

CdtB is the most conserved subunit of CDT, sharing at least 48% of identical amino acids in the respective positions among the identified CDTs. More particularly, putative catalytic and metal binding residues are strictly conserved between CdtBs and the other members of the EEP family [75], strongly suggesting their implication in CdtB activity. The EEP family includes nucleases and PIP phosphatases, DNase I and synaptojanin being among the most well characterized. While the structural similarities between CdtB and DNase I are well established [13], the functional correlation partly relies on the plasmid digestion assay. However, this test does not seem to be adapted for CdtB, which exhibits a very weak activity compared to DNase I. In addition, discrepancies have been reported concerning the activity of many CdtB mutants. Indeed, some recombinant CdtBs mutated in catalytic or metal binding residues were shown to exhibit an activity close to the WT or to recombinant CdtA or CdtC, devoid of any catalytic domain [40,76,77,88,89]. On the other hand, CdtB-related DNA damage has been widely described in cellular systems, either by direct visualization of genomic DNA fragmentation or through DDR activation. In these cases, mutational analyses confirmed that the catalytic and metal binding residues are essential for CDT-mediated DNA damage induction, cell cycle arrest and apoptosis. Therefore, there is a missing piece between the CdtB-induced genotoxicity observed in cells and the lack of biochemical evidence to definitively demonstrate its nuclease activity. One can speculate that plasmidic nude DNA is an inappropriate substrate for CdtB, or that a cellular factor is essential for CdtB activity. Future studies will be necessary to elucidate this issue.

Structural comparison also revealed similarities between CdtB and the IP5P domain of synaptojanin [10]. To corroborate this, CdtB has been shown to dephosphorylate PIP_3_ in vitro [77] and this activity depends on the catalytic and metal binding residues. Thus, one catalytic site seems to bear the two CdtB activities, complicating elucidation of the role of each activity by mutational analyses. Therefore, investigators have to explore other approaches to uncouple nuclease and phosphatase activities. Based on structural comparison, three putative DNA binding residues have been identified [13]. However, two of them, namely R117 and R144, were also suspected to be involved in PIP_3_ binding by docking analyses [28]. The third one, N201, is strictly conserved among the EEP family members. Therefore, its mutation may also influence the phosphatase activity. A163 was predicted to bind PIP substrates, but was not clearly shown to be involved in phosphatase activity, whereas its mutation diminished DDR activation in cells [28]. To date, efforts performed to specifically alter CdtB activity are still not convincing, and novel strategies must be considered to successfully reach this goal. In conclusion, deciphering genotoxin mechanisms can help to pharmacologically target their activities and better understand their physiopathology [103]. Therefore, studying and comparing different CDTs mode of action will be a first step to get more insights into the effect of this toxin on human and animal health.

## Figures and Tables

**Table 1:** Effects of mutations in CdtBs of different bacteria compared to their respective WT forms: increased (↗), unchanged (≈), decreased (↘) or loss of activity/effect (Ø). For intracellular localization (Intracell. loc.), either correct (OK), mislocated (mis) or non-detected (Ø) localization is noted. For some experiments, details are provided at the table end. CRAC: cholesterol recognition amino acid consensus sequence.

Type of Residues	CDT	Mutation	DNaseI Residue	In Vitro Activity			Cellular and Vivo Effects						Other effects	References
				Plasmid Digestion	PIP_3_ Digestion		Cellular Distension	Cell cycle Block	Cellular Death	DNA Damage	Intracell. loc.			
**Catalytic**	Ecol-II	E86A	78	↘			↘	≈						[73]
	Hduc	V118E		Ø				Ø						[13]
	Aact	H160Q	134	↘	↘			Ø						[77]
	Aact	H160G		≈	↗				↘					[40]
	Aact	H160A							Ø					[92]
	Aact	H160A						Ø	Ø		OK			[64]
	Cjej	H152Q					Ø	Ø		Ø				[12]
	Cjej	H152A						Ø		Ø				[93]
	Ecol-I	H153A								Ø				[90]
	Ecol-I	H153A								Ø				[25]
	Ecol-I	H153A								Ø				[27]
	Ecol-I	H153A								Ø				[51]
	Ecol-I	H153A		≈						Ø				[89]
	Ecol-II	H154A		Ø			Ø	Ø						[73]
	Ecol-II	H154A		Ø				Ø						[84]
	Hpar	H161Q		Ø						Ø				[94]
	Styp	H160Q						Ø		Ø				[57]
	Styp	H160Q											Ø ^1^	[14]
	Cjej	D222A	212				Ø	Ø	Ø	Ø				[95]
	Cjej	D222A						Ø		Ø				[96]
	Ecol-II	D229A		Ø			Ø	Ø						[73]
**Catalytic**	Aact	H274Q	252	≈	↘			Ø						[77]
	Aact	H274A							↘					[97]
	Aact	H274A					Ø	Ø	Ø					[92]
	Aact	H274A											↘ ^2^	[98]
	Ecol-II	H261A		Ø			Ø	Ø						[73]
	Hhep	H265L		Ø					Ø					[76]
	Hhep	H265L					Ø			Ø	OK		↘ ^3^	[36]
	Hhep	H265L					Ø	Ø	Ø					[99]
**Patients**	Aact	H281R		↗			↗	↗		↗				[87]
	Aact	H281A		≈			Ø	Ø		↘				[87]
	Aact	H281K					↗	↗						[87]
	Aact	H281D					↘							[87]
	Aact	Δ116-188		↗	Ø				Ø	Ø				[88]
**Metal binding**	Hhep	N27I		≈					Ø					[76]
	Hhep	E60V	39	≈					Ø					[76]
	Aact	D199S	168	↘	↘			Ø						[77]
	Aact	D199G		≈	↘				Ø					[40]
	Cjej	D185S					Ø	Ø		Ø				[12]
	Styp	D195S						Ø		Ø				[57]
	Ecol-II	D260R	251	Ø			Ø	Ø						[73]
	Hduc	D273R						Ø		Ø				[69]
	Hduc	D273R								Ø			Ø ^4^	[100]
	Hduc	D273R								Ø			Ø ^5^	[101]
	Hduc	D273R		≈						Ø				[89]
	Aact	D199A	168					Ø	Ø		OK			[64]
		D273A	251											
**Substrate Binding**	Aact	R117A	41	↘	↘			Ø						[77]
	Aact	R117A		≈	↘			Ø						[28]
	Aact	R144A	111										↘ ^3^	[42]
	Aact	R144A		↗	↘			Ø		Ø				[28]
	Aact	N201A	170										↘ ^3^	[42]
	Aact	N201A		Ø	↘			Ø						[28]
**Substrate Binding**	Aact	Q35A						Ø	Ø		OK			[64]
		N201A	170											
		Y239A												
	Aact	R117A	41	↗	↘			Ø						[28]
		R144A	111											
		N201A	170											
	Hduc	R117A	41	↘				Ø						[13]
		R144A	111											
		N201A	170											
**Putative Phosphatase Activity**	Aact	A163R											↘ ^3^	[42]
	Aact	A163R		Ø	↘			↘ ≈ ^6^		Ø				[28]
	Aact	F156I		↗	↘			Ø						[28]
		T158I												
	Aact	F156I		↗	↘			Ø						[28]
		T158I												
		A163R												
	Aact	Y239R		↗	↘			Ø						[28]
	Aact	Y239R		Ø	Ø			Ø						[28]
		A240I												
	Aact	D244G		Ø	Ø			Ø						[28]
		H246L												
	Aact	Y239R A240I D244G H246L		↗	↘			Ø						[28]
**Multiple**	Hduc	H160Q D199S		Ø				Ø						[13]
	Aact	R117A H160A D199S H274A		Ø	Ø				Ø					[102]
**CRAC**	Aact	V104P			≈			Ø	Ø		Ø		↘ ^7^ ↘ ^8^	[62]
	Aact	Y105P			≈			Ø	Ø		Ø		↘ ^7^ ↘ ^8^	[62]
	Aact	Y107P			≈			Ø	Ø		Ø		↘ ^7^ ↘ ^8^	[62]
	Aact	R110P			≈			≈	≈		OK		↗ ^7^ ≈ ^8^	[62]
**Transport**	Aact	Δ48-124							Ø		mis			[67]
	Aact	Δ48-124 + SV40 T							≈		OK			[67]
	Aact	Δ114-124					Ø	Ø	Ø		mis			[67]
	Aact	Δ114-124 + SV40 T					≈	≈	≈		OK			[67]
	Aact	Δ114-124							Ø					[92]
	Aact	R126S R127T						↘	≈		OK			[64]
	Ecol-II	Δ195-210		≈				Ø			mis		≈ ^8^	[68]
	Ecol-II	Δ253-269		≈				≈ Ø ^9^			mis		≈ ^8^	[68]
	Ecol-II	Δ195-210 Δ253-269		≈				Ø					≈ ^8^	[68]
	Aact	R189S R190T						↘	↘		mis			[64]

^1^ Adverse effects in mice. ^2^ Epithelial damage on rat gingival explants. ^3^ Inflammation marker induction. ^4^ Autophagy induction. ^5^ Epstein Barr virus reactivation. ^6^ Cycle block reduced in non-adherent cells and unchanged in adherent cells. ^7^ In vitro cholesterol binding ability. ^8^ Cellular binding ability. ^9^ Cycle block unchanged upon CdtB electroporation and abolished for holotoxin.
